# Reassessing the Biological Significance of Congenital Melanocytic Nevi

**DOI:** 10.5826/dpc.1003a68

**Published:** 2020-07-09

**Authors:** Giuseppe Argenziano, Stefano Caccavale, Gabriella Brancaccio, Elvira Moscarella, Vincenzo Piccolo, Aimilios Lallas

**Affiliations:** 1 Dermatology Unit, University of Campania, Naples, Italy; 2 First Department of Dermatology, Aristotle University, Thessaloniki, Greece

## Introduction

In the context of this editorial on congenital melanocytic nevi (CMN), the following points will be discussed:

1. Medium and large CMN are hamartomas present at birth, but many small CMN can appear later, within the first decade of life.
2. Most CMN of any size will persist throughout a lifetime, but some, especially those located on special areas, including nails and acral sites, might disappear as a result of spontaneous involution.
3. If there is any precursor lesion of melanoma, then CMN is entitled to be the most important one.

## The CMN Family

**Figure 1:**
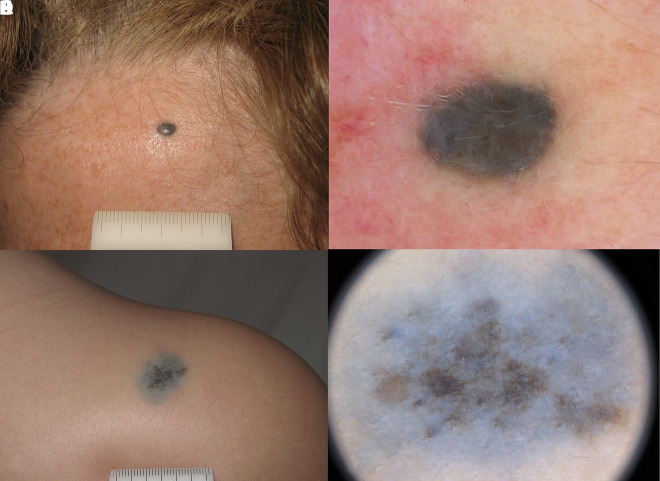
(A,B) Clinical and dermoscopic images of a blue nevus. (C,D) Nevus of Ito on the shoulder of a young boy.

Traditionally, CMN are classified into 2 groups, namely, CMN blue type and CMN non-blue type [1]. Of the first group, blue nevi are the most frequent ones (Figure 1, A and B); other dermal melanocytoses belonging to this group of CMN include mongolian spot, nevus of Ota, and nevus of Ito (Figure 1, C and D).

CMN of the non-blue family are also defined as superficial CMN to point out the main difference from blue-type CMN, which are typified by a more deeply situated proliferation of dendritic melanocytes. Superficial CMN are traditionally subdivided into the following 3 groups based on the diameter they are expected to attain in adulthood: small CMN, <2 cm; medium CMN, 2–20 cm; and large CMN, >20 cm. This subdivision is arbitrary, but it is based on the fact that the risk for melanoma increases with the increasing size of CMN.

**Figure 2:**
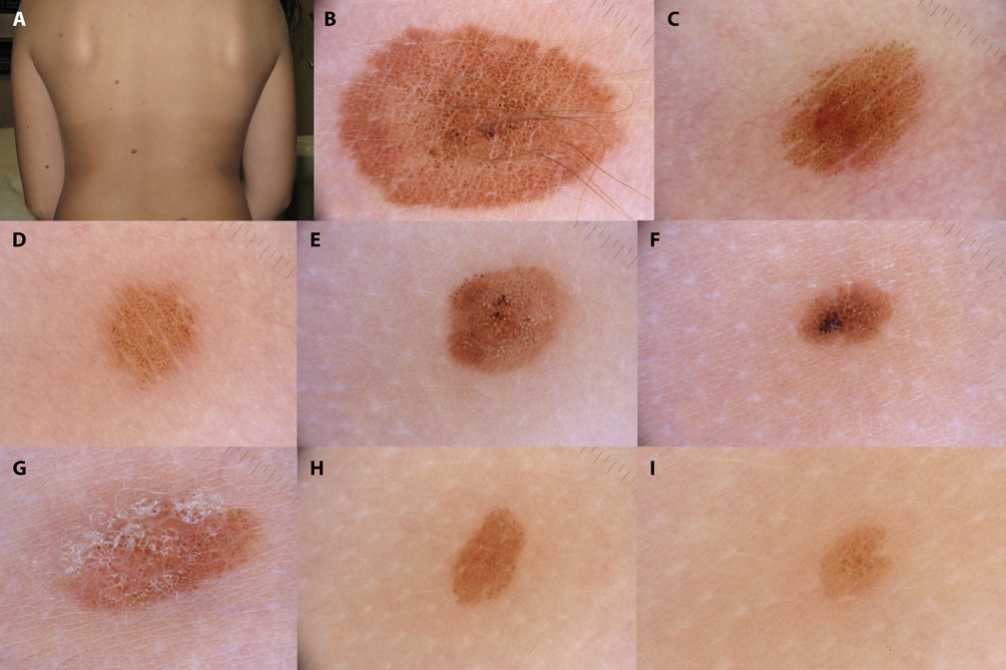
A 10-year-old girl with multiple nevi. Based on the history, the only “true” congenital melanocytic nevus (CMN) is the one depicted in (B), whereas the other nevi shown (C-I) appeared within the first 2–3 years of life. However, based on morphology, all of them show a globular pattern; thus the hypothesis is that all these nevi belong to the same spectrum of lesions, namely, CMN.

Medium and large CMN are hamartomas typified by a benign proliferation of melanocytes and keratinocytes [2,3]. They are always present at birth, whereas small CMN can frequently appear later, usually before puberty [3]. These nevi are commonly classified as early acquired melanocytic nevi (AMN), but in our estimation they essentially belong to the family of CMN. This is mainly based on their similarity to “true congenital” melanocytic nevi in terms of morphological features and evolutional behavior (Figure 2).

**Figure 3:**
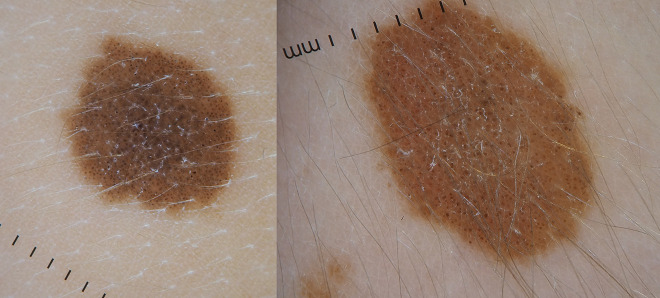
Small congenital melanocytic nevi dermoscopically typified by a globular pattern.

**Figure 4:**
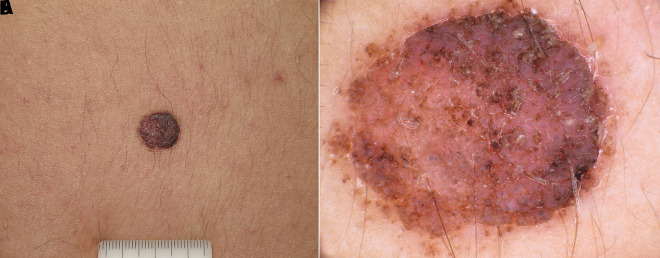
(A,B) Clinical and dermoscopic images of a dermal congenital melanocytic nevus on the trunk with its papillomatous surface.

Morphologically, most small CMN (either present at birth or appearing in early childhood) are clinically flat or slightly raised pigmented lesions dermoscopically typified by a globular pattern (Figure 3). The presence of brown globules at the periphery is the hallmark of the growing phase of the lesion, which tends to become stable as soon as the globules disappear [3–5]. Over a variable period of time, usually years, small CMN become more elevated and acquire a papillomatous (usually on the trunk) (Figure 4) or smooth (usually on the face) surface. Dermoscopically, brown globules at the periphery disappear, while a cobblestone pattern becomes visible throughout the lesion.

**Figure 5:**
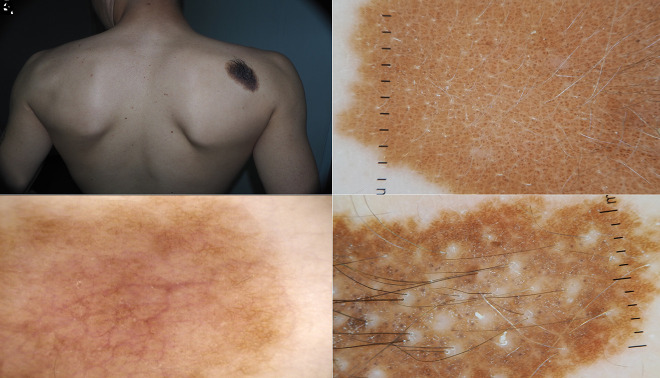
(A) Intermediate size congenital melanocytic nevus (CMN) with its verrucous surface intermingled with terminal hairs. Dermoscopically, medium and large CMN may exhibit (B) a globular pattern, (C) a reticular pattern, or (D) a combination of both.

**Figure 6:**
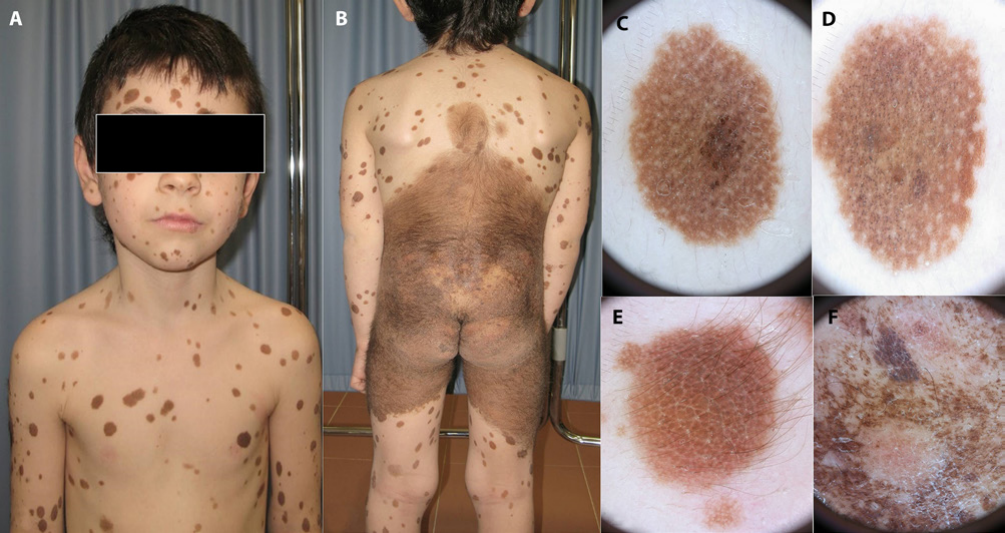
A boy with a giant congenital melanocytic nevus (CMN) associated with multiple smaller CMN (satellitosis).

In medium and large CMN, the keratinocytic proliferation is usually seen as a verrucous surface intermingled with terminal hairs. Dermoscopically, medium and large CMN may exhibit a globular pattern, a reticular pattern, or a combination of both (Figure 5). Most large CMN are confined to the skin, but in rare cases they may involve the central nervous system configuring the so-called neurocutaneous melanosis. This is a potentially life-threatening condition that usually occurs when a large CMN is accompanied by multiple smaller CMN (satellitosis) and mostly in bathing trunk nevi (Figure 6) [6]. In this clinical context a brain nuclear magnetic resonance is usually indicated within the first year of life [7].

## The Life Cycle of CMN

As mentioned above, small CMN usually tend to persist over time. Especially when located on the head-neck, trunk, and limbs, small CMN undergo progressive maturation within the dermis, thus evolving into intradermal nevi. In our estimation, the great majority of dermal melanocytic nevi in adulthood belong to the group of small CMN. This is because most of AMN do not show a tendency to maturation. They appear usually after puberty as a superficial, junctional, or compound proliferation of melanocytes, growing for a variable period of time and finally going through spontaneous involution [8,9].

**Figure 7:**
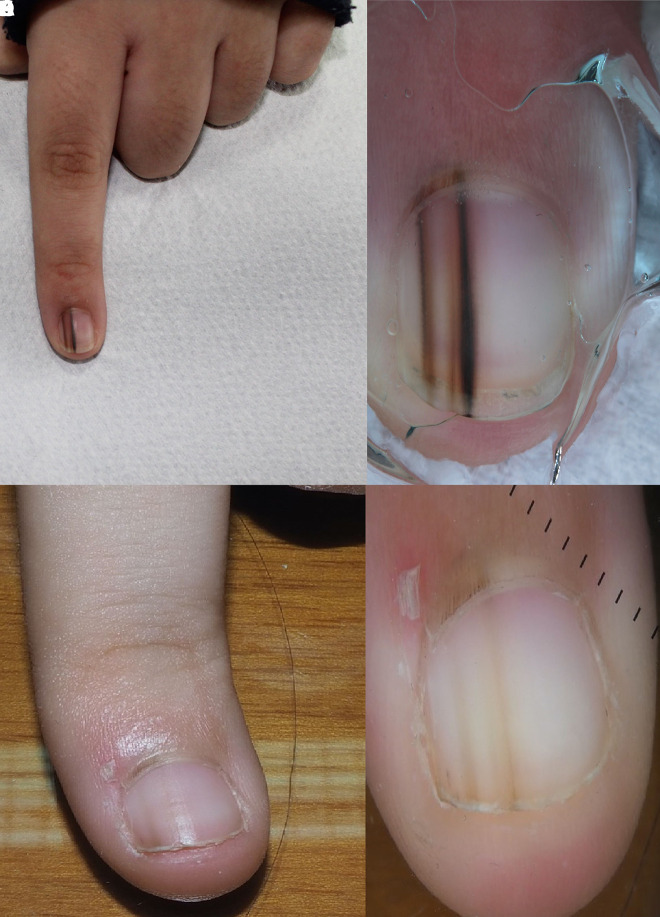
(A,B) Clinical and dermoscopic images of a congenital melanocytic nevus of the nail matrix, undergoing spontaneous involution after 2 years (C,D).

Although most small CMN tend to persist over time, there are instances in which CMN are prone to disappear. This is particularly the case in special areas including nails (Figure 7) and acral sites where CMN may undergo spontaneous involution after a variable period of time, usually years [10–14].

It is unknown why some CMN undergo maturation and some disappear. A possible explanation might be related to the depth level of the skin in which the nevus is mainly proliferating. CMN of the head-neck region, trunk, and limbs are usually compound nevi histopathologically, whereas CMN of the nails and acral sites are more of the junctional type [15]. It is possible that dermal melanocytes may tend to undergo maturation while epidermal melanocytes are more prone to disappear after a variable period of time, similarly to AMN. The latter are also typified by a junctional proliferation of melanocytes and they also usually undergo spontaneous involution over time [10–16].

## CMN and Melanoma Risk

The dogmatic knowledge about the risk of melanoma in CMN states that the larger the nevus, the higher the chance of melanoma development within a preexisting CMN. Most of the studies assessing this risk included medium and large CMN [17–21]. In one of the largest studies of more than 6,500 patients with medium and large CMN, 0.7% of patients developed melanoma at a median age of 7 years [22]. However, the risk of developing melanoma was by far highest in CMN 40 cm or more in diameter. In the latter group, the risk may reach 10% [22]. If we compare these percentages to the risk of melanoma within an AMN (recently calculated as approximately 1 out of 200,000 AMN) [23], we can conclude that CMN is by far the most important melanoma precursor.

Melanoma risk in small CMN has not yet been calculated, perhaps because of the limitations in discriminating between small CMN and AMN. In a recent study by our group, however, we reported that at our institution most of the melanomas arising within a CMN (78%) were developing in the context of a small CMN (unpublished data). This seems paradoxical, but it can be easily explained by the respective numbers of small, medium, and large CMN seen in routine practice. Assuming that the risk of melanoma in a large CMN is 10%, we have to screen 10 large CMN to find 1 melanoma. In our institution, we screen a mean of 2 new patients with large CMN per year; thus we need 5 years to find 1 melanoma. Assuming that the risk of melanoma in a small CMN is much lower than 10%, still the chance of finding one is much higher because of the much higher incidence of small CMN in the clinical routine.

**Figure 8:**
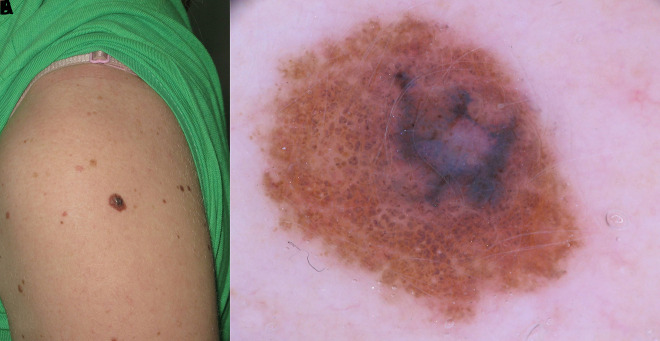
Clinical and dermoscopic images of a melanoma developing in association with a small congenital melanocytic nevus on the arm of a teenager.

Pediatric melanoma is, in general, an extremely rare disease. In a recent systematic review, we found 2 important issues to mention (unpublished data). First, the majority (about 55%) of pediatric melanomas (defined as melanoma occurring in patients aged 18 years or less) develop as CMN-associated melanoma (Figure 8). This is in contrast to what is happening in adults, in whom at least 70% of melanomas arise de novo. The second is related to the mortality for pediatric melanoma, which is higher for nevus-associated melanoma than for de novo melanoma, especially for children within the first decade of life. This difference was not attributed to Breslow thickness, which was similar in both groups. Thus, the only possible explanation is that at least a certain percentage of lesions that were reported as de novo melanoma were instead benign melanocytic tumors. This underlines once more the problem of morphologically difficult lesions in children, as already reported by Leman et al [24]. If this is true, then the percentage of pediatric melanomas that develop on CMN is even higher than 55%.

## Conclusions

CMN are much more relevant lesions than previously considered. They are more common than usually reported because early AMN might also belong to the group of CMN. They can persist or disappear based on their localization on different body areas and, much more frequently than AMN, they can be found in association with melanoma, in both children and adults. Small CMN, although traditionally considered as not requiring special attention, might be the most frequent melanoma precursor in terms of absolute numbers. This does not mean that the risk of melanoma on small CMN is high, but that the majority of nevi on which melanoma develops belong to the category of small CMN [25].

## References

[1] Moscarella E, Piccolo V, Argenziano G (2013). Problematic lesions in children. Dermatol Clin.

[2] Schaffer JV (2015). Update on melanocytic nevi in children. Clin Dermatol.

[3] Errichetti E, Patriarca MM, Stinco G (2017). Dermoscopy of congenital melanocytic nevi: a ten-year follow-up study and comparative analysis with acquired melanocytic nevi arising in prepubertal age. Eur J Dermatol.

[4] Cotton CH, Goldberg GN (2019). Evolution of congenital melanocytic nevi toward benignity: a case series. Pediatr Dermatol.

[5] Odorici G, Longhitano S, Kaleci S (2020). Morphology of congenital nevi in dermoscopy and reflectance confocal microscopy according to age: a pilot study. J Eur Acad Dermatol Venereol.

[6] Alikhan A, Ibrahimi OA, Eisen DB (2012). Congenital melanocytic nevi: where are we now? Part I: clinical presentation, epidemiology, pathogenesis, histology, malignant transformation, and neurocutaneous melanosis. J Am Acad Dermatol.

[7] Kinsler VA, Aylett SE, Coley SC, Chong WK, Atherton DJ (2001). Central nervous system imaging and congenital melanocytic naevi. Arch Dis Child.

[8] Vezzoni R, Conforti C, Vichi S (2020). Is there more than one road to nevus-associated melanoma?. Dermatol Pract Concept.

[9] Pizzichetta MA, Massone C, Grandi G, Pelizzo G, Soyer HP (2006). Morphologic changes of acquired melanocytic nevi with eccentric foci of hyperpigmentation (“Bolognia sign”) assessed by dermoscopy. Arch Dermatol.

[10] Haenssle HA, Blum A, Hofmann-Wellenhof R (2014). When all you have is a dermatoscope—start looking at the nails. Dermatol Pract Concept.

[11] Marghoob AA (2002). Congenital melanocytic nevi: evaluation and management. Dermatol Clin.

[12] Strauss RM, Newton Bishop JA (2008). Spontaneous involution of congenital melanocytic nevi of the scalp. J Am Acad Dermatol.

[13] Lee NR, Chung HC, Hong H, Lee JW, Ahn SK (2015). Spontaneous involution of congenital melanocytic nevus with halo phenomenon. Am J Dermatopathol.

[14] Nath AK, Thappa DM, Rajesh NG (2011). Spontaneous regression of a congenital melanocytic nevus. Indian J Dermatol Venereol Leprol.

[15] Zalaudek I, Longo C, Ricci C, Albertini G, Argenziano G, Marghoob AA (2012). Classifying melanocytic nevi. Nevogenesis.

[16] Kinsler VA, O’Hare P, Bulstrode N (2017). Melanoma in congenital melanocytic naevi. Br J Dermatol.

[17] Illig L, Weidner F, Hundeiker M (1985). Congenital nevi less than or equal to 10 cm as precursors to melanoma: 52 cases, a review, and a new conception. Arch Dermatol.

[18] Rhodes AR, Melski JW (1982). Small congenital nevocellular nevi and the risk of cutaneous melanoma. J Pediatr.

[19] Rhodes AR, Sober AJ, Day CL (1982). The malignant potential of small congenital nevocellular nevi: an estimate of association based on a histologic study of 234 primary cutaneous melanomas. J Am Acad Dermatol.

[20] Berg P, Lindelöf B (2003). Congenital melanocytic naevi and cutaneous melanoma. Melanoma Res.

[21] Tannous ZS, Mihm MC, Sober AJ, Duncan LM (2005). Congenital melanocytic nevi: clinical and histopathologic features, risk of melanoma, and clinical management. J Am Acad Dermatol.

[22] Krengel S, Hauschild A, Schäfer T (2006). Melanoma risk in congenital melanocytic naevi: a systematic review. Br J Dermatol.

[23] Tsao H, Bevona C, Goggins W, Quinn T (2003). The transformation rate of moles (melanocytic nevi) into cutaneous melanoma: a population-based estimate. Arch Dermatol.

[24] Leman JA, Evans A, Mooi W, MacKie RM (2005). Outcomes and pathological review of a cohort of children with melanoma. Br J Dermatol.

[25] Caccavale S, Calabrese G, Mattiello E Cutaneous melanoma arising in congenital melanocytic nevus: a retrospective observational study. Dermatology.

